# Multispectral regulation of chromatic acclimation by integration of the Rca system and the conserved *dpx* operon

**DOI:** 10.1128/jb.00023-26

**Published:** 2026-07-15

**Authors:** Lisa B. Wiltbank-Chau, James I. Cohen, Devin S. Burdett, Brighton Larson, Prapti Khadka, Tiffani R. Peterson, David M. Kehoe

**Affiliations:** 1 Department of Microbiology, Weber State University 6181, Ogden, Utah, USA; 2 Department of Botany and Plant Ecology, Weber State University 6181, Ogden, Utah, USA; 3 Department of Biology, Indiana University 1772, Bloomington, Indiana, USA; The Ohio State University, Columbus, Ohio, USA

**Keywords:** cyanobacteria, cyanobacteriochromes, chromatic acclimation, photosensing, signal transduction, phycobilisomes, *Fremyella diplosiphon*

## Abstract

Type III Chromatic Acclimation (CA3) in *Fremyella diplosiphon* has traditionally served as a model for cyanobacterial acclimation to red and green light. CA3 is controlled by the red-green responsive Rca phosphorelay system, including the cyanobacteriochrome RcaE. However, regulation of pigmentation persists in the absence of RcaE, which is attributed to the Cgi system. Nothing is known about how the Cgi regulatory system senses light color to differentially regulate pigment expression. DpxA is a teal/yellow-sensing cyanobacteriochrome that regulates cell pigmentation through an unknown regulatory pathway. Here, we show that DpxA and RcaE collectively control the vast majority of chromatic acclimation across the visible spectrum, implicating DpxA as the likely sensor for the Cgi system. The genetic context of DpxA is identified as part of a 3-gene operon alongside a response regulator (DpxB) and a hybrid histidine kinase (DpxC). The impact of the Dpx proteins is modulated by the RcaE-mediated genetic background, suggesting an integrated sensory response by the Dpx and Rca pathways. Beyond mechanistic insights, phylogenomic analysis reveals that the *dpx* operon is conserved across distantly related cyanobacterial species, including those incapable of CA3. These findings suggest the *dpx* operon was distributed via horizontal gene transfer, likely providing cell adaptation to diverse environmental niches through regulation of processes beyond color acclimation.

**IMPORTANCE:**

The ability to accurately sense and integrate multiple environmental cues is fundamental to bacterial behavior and survival. Type III Chromatic Acclimation (CA3) in *Fremyella diplosiphon* is an ideal model for signal integration because of its well-defined light cues, numerous predicted photoreceptors, and reliable physiological output. This study reveals that CA3 is governed by an interconnected, multispectral sensory network. We demonstrate that the conserved *dpxABC* operon—and its sensor DpxA—integrates with the Rca pathway into a coordinated acclimation response. Furthermore, the *dpx* operon is conserved across phylogenetically distant cyanobacteria, suggesting physiological benefits beyond CA3. These findings underscore the complexity of prokaryotic light signal integration, wherein multiple cues are synthesized into cohesive physiological responses.

## INTRODUCTION

Cyanobacteria are experts at adaptation and colonization, surviving in some of the harshest environments on Earth (1, 2). Their success is dependent upon effectively utilizing energy from the sun to drive photosynthesis, a process that is disturbed by fluctuations in light conditions. To compensate for variations in light quality and quantity, many cyanobacteria have evolved mechanisms that tune photon capture to the available environmental light in a process known as chromatic acclimation (3, 4). At least seven types of chromatic acclimation have been described (5).

The freshwater cyanobacterium *Fremyella diplosiphon* (*Tolypothrix* sp. *7601* or *Calothrix* sp. *7601*) is a model organism for red-green acclimation called Type III Chromatic Acclimation (CA3). During CA3, cells reorganize the light-harvesting phycobilisomes, antenna structures that increase surface area for photon capture and funnel energy to chlorophyll in the photosynthesis reaction centers (6–8). Phycobilisome restructuring maximizes the use of incident irradiation. In *F. diplosiphon*, the phycobilisome is fan-shaped and hemidiscoidal, with a core of allophycocyanin (AP) and six rods which emanate from the core (9, 10). In red light, the outer rods of the phycobilisomes are made primarily of the green-pigmented phycocyanin (PC), while in green light, the outer portion of the rods are instead composed of red-pigmented phycoerythrin (PE) (3, 11–13). This change in the outer rods is due to gene expression regulation in each light color.

*F. diplosiphon* has three known systems to regulate the composition of photosynthetic light-harvesting antenna structures in response to light color. The best-characterized of the three systems is the Rca phosphorelay pathway, which includes 3 proteins: RcaE, RcaF, and RcaC. RcaE is a sensor kinase that senses red and green and activates a signal transduction pathway that regulates PE and PC gene expression (14, 15). RcaE is a member of GAF-containing photoreceptors called cyanobacteriochromes (16, 17), and its green-red photocycle has been elucidated (18, 19). In red light, RcaE autophosphorylates and activates a phosphorelay including the response regulators (RRs) RcaF and RcaC (20, 21). RcaC is a transcription factor that, when phosphorylated, binds to the promoters of PE and PC-related genes to repress PE and activate PC (22, 23), resulting in green cells that contain high levels of PC. In green light, RcaE does not phosphorylate RcaF or, in turn, RcaC, meaning that PE is not repressed and PC is not activated, resulting in high levels of PE and red-colored cells. The Rca system is the main mode of control for CA3 and is the only system in *F. diplosiphon* that is known to both repress PE and activate PC in response to light color.

The second system that acclimates *F. diplosiphon* to red and green light is the Cgi system, which represses PE in red light but has no effect on PC. The activity of the Cgi system becomes apparent in the absence of the Rca pathway, where red-green regulation of PE is still observed (24, 25). It is unknown how the Cgi system senses red and green light or whether the Cgi system maximally responds to red and green light, as no action spectrum for the Cgi system has been published. The Cgi system represses PE in red light by transcription attenuation involving the 5’ leader sequence of *cpeC* (26). The transcription initiation factor IF3a represses PE in a red-green light-dependent manner in the absence of RcaE (27), which may indicate that IF3a has a role in the Cgi system, but that has yet to be demonstrated.

A third system of pigmentation regulation involves DpxA as a sensor and appears to be unique from other known CA3 systems in that it regulates PE in response to teal and yellow light (28). PE levels are very high in blue light but PE abundance is repressed in yellow light. DpxA is a cyanobacteriochrome that senses yellow and teal light. The Dpx system is proposed to be a fine-tuning mechanism in the green region of the spectrum, where RcaE is inactive (29). DpxA is the sensor in this system, but little is reported about DpxA and its mechanism of action, genome context, and phylogeny. In this study, we sought to uncover more about the components and role of the Dpx system in CA3 and to begin to examine whether Dpx and Cgi components are part of the same pathway.

## MATERIALS AND METHODS

### Bacterial strains, plasmids, and growth conditions

Wild type in this study was the *F. diplosiphon* shortened filament mutant strain SF33 (derived from UTEX 481/PCC 7601) (30). Cultures were grown in BG-11 medium, as described (24), in light irradiances of 12–15 µmol photons m^−2^ s^−1^. Custom-built LED panels contributed red light (https://www.digikey.com/, 160-1415-2-ND), green light (https://www.digikey.com/, 754-1099-2-ND), blue light (https://www.digikey.com/, 516-2672-1-ND), and yellow light (https://www.digikey.com/, 516-2673-1-ND). Solux 4700 Kelvin halogen lamps (Eiko Ltd., Q50MR16/CG/47/36) were used for white light.

The *dpxA and rcaC* mutants were those described previously (28). *dpxB, dpxC,* and *rcaE* single and double null mutant strains were created using a previously described method (28). Primers are listed in Table 1, and the cloning vector used for deletion was pJCF276 (31). The deletion constructs of *dpxB, dpxC,* and *rcaE,* and double mutants were confirmed by sequencing. Wild-type *F. diplosiphon* was transformed by triparental mating, and mutants were obtained by two selection steps: first with neomycin, then by counterselection with sucrose as previously described (31). Three independent colonies of each null mutant were selected and tested by PCR amplification and sequencing. *rcaEdpxA* double mutants were complemented by cloning *dpxA* and its native promoter into the shuttle vector pPL2.7 (30), as previously described (28). Three independent growth experiments were conducted for each cell type and light condition, and a representative photograph and scan are presented for each. Transposon mutants were found in a genetic screen as described in a previous publication (27).

**TABLE 1:** Primers used in this study

Purpose	Plasmid	Primer sequence
Gene deletions		
*rcaE* deletion	pJCF276rcaEdel	5'CGACGCCCATGGTCGCATCGATCTGCACACAGAT3'
		5'CGACAGATAGTCAGCCCTAAACCAGTAAATCACAAG CAGCAATATTCATAT3'
		5'GGTGCGCCATGGGGAGCGAAGCGAAGTGACTCA3'
		5'ATATGAATATTGCTGCTTGTGATTTACTGGTTTAGGGC TGACTATCTGTCG3'
*dpxB* deletion	pJCF276dpxBdel	5'CGACGCCCATGGGATTGCCTGGATTTCTGAGAATGAT3'
		5'CAGAGGAGGTGGGTGGATGAG GCCTGTTTAGGAA GTCAGTGACA3'
		5'ACGCCCATGGGGAAGGGTGCAAGCAGTTGC3'
		5'TGTCACTGACTTCCTAAACAGGCCTCATCCACCCA CCTCCTCTG3'
*dpxC* deletion	pJCF276dpxCdel	5'CGACGCCATGGGGCGATCGCATCTGGTATATTCA3'
		5'GTTTTAAGAGAACATGAGCAAAAACAGCTCCTTGG TATCCGAGAATTC3'
		5'CAAGCCATGGGGTTGATGCGTAGTGAGATGCAG3'
		5'TTTTTGCTCATGTTCTCTTAAAACTAGGAGAGTATC ATCTTCAGAATCTTCAACAATTAG3'
RT-PCR analysis		
*cpeSlike* forward		5'CCTTCTCATCTGAGATTCGCATG3'
*dpxA* forward		5'GCGATGATGGTGCTGGCTT3'
*dpxB* forward		5'CTCGTGATGGAGTAGAAGCGCTAG3'
*dpxC* reverse		5'ATGGCCGCAACCGCAATATC3'
RNA blot probe		
*cpeC*		5'AGCAGAAACAATCATTCGGGCA3'
		5'TGCTGTTGGTGTGCTTTGAATC3'

### Reverse transcription PCR

RNA was extracted from wild-type *F. diplosiphon* cells as previously described (24). 100 ng RNA was used in the Qiagen QuantiTect Reverse Transcription Kit according to the protocol provided. Primers used are listed in Table 1. NEB Phusion polymerase was used in the final PCR reaction with an extension time of 1 min and 45 s.

### RNA blots

RNA blots were performed as previously described (24). 10 µg of RNA was used for each lane. Probes were made using the primers in (26). Bands were quantified using ImageQuant software and normalized to ribosomal bands. Quantification represents the average of three independent experiments.

### Spectral measurements

A Beckman DU640B spectrophotometer measured the spectrum of intact *F. diplosiphon* cultures from 375 nm–725 nm. Cells were harvested in late log phase at an A_750_ of 1.0–1.2 as previously described (28).

### Patterns of evolution of Dpx

Sequences similar to DpxA were identified by searching the known sequence in *F. diplosiphon* against the National Center for Biotechnology Information (NCBI) genome database using BLAST (32), specifically BLASTP. Proteins' similarity to Dpx proteins were downloaded in FASTA format and aligned using the multiple sequence alignment Clustal Omega (33). The identity between proteins was determined by a pairwise identity matrix using the Percent Identity Matrix feature, also available through the Clustal Omega program.

Additionally, the 16S rRNA region was downloaded from NCBI for 694 species of cyanobacteria. These sequences were aligned with MAFFT v7.407 using the G-INS-i method (34). Subsequently, the phylogeny of 16S was resolved using IQ-TREE (35), with ModelFinder employed to identify the most appropriate model with Bayesian, Akaike, and corrected Akaike information criteria (BIC, AIC, and AICc, respectively), using 1,000 ultrafast bootstrap replicates and the bnni option to reduce the possibility of overestimating branch support. Trees from different models were compared, and the BIC tree was employed to recognize the phylogenetic distribution of the *dpx* genes across cyanobacteria.

## RESULTS

### Wild-type *F. diplosiphon* exhibits both red-green and yellow-blue acclimation systems

**Fig 1:**
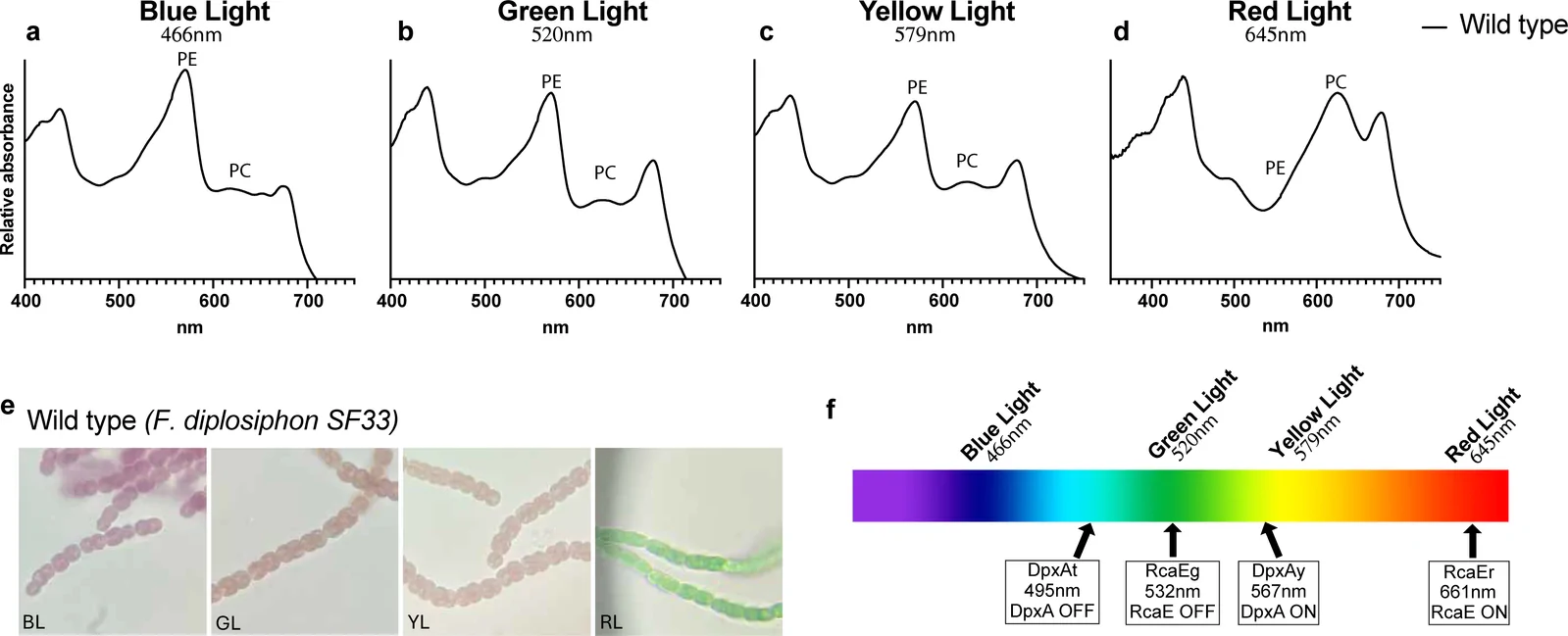
Representative whole-cell absorbance scans of *F. diplosiphon* SF33 (wild type) grown in (**a**) blue 460 nm, (**b**) green 520 nm, (**c**) yellow 579 nm, and (**d**) red 645 nm light at 12 µmol photons m^−2^ s^−1^. PE and PC pigment peaks are indicated on the diagram. (**e**) Microscopy images of cells grown in the colors indicated. (**f**) Maximum absorbance of DpxA and RcaE based on previously published biochemical assays.

Wild-type *F. diplosiphon* cells were grown in blue, green, yellow, or red light. Comparing cells grown in blue light versus yellow light, wild-type cells were brown in yellow light and brighter red in blue light, which is indicative of blue-yellow acclimation previously shown for DpxA-controlled acclimation (Fig. 1a, c, and e). When comparing the pigmentation of cells grown in red light versus green light, cells display the classic red-to-green shift, which is indicative of RcaE-controlled acclimation (Fig. 1b, d, and e). Wild-type cell pigmentation matches the wavelengths of *in vitro* maximal absorbance of DpxA and RcaE in their “on” and “off” states (Fig. 1f).

### Loss of RcaE and DpxA abolishes chromatic acclimation across the visible spectrum

**Fig 2:**
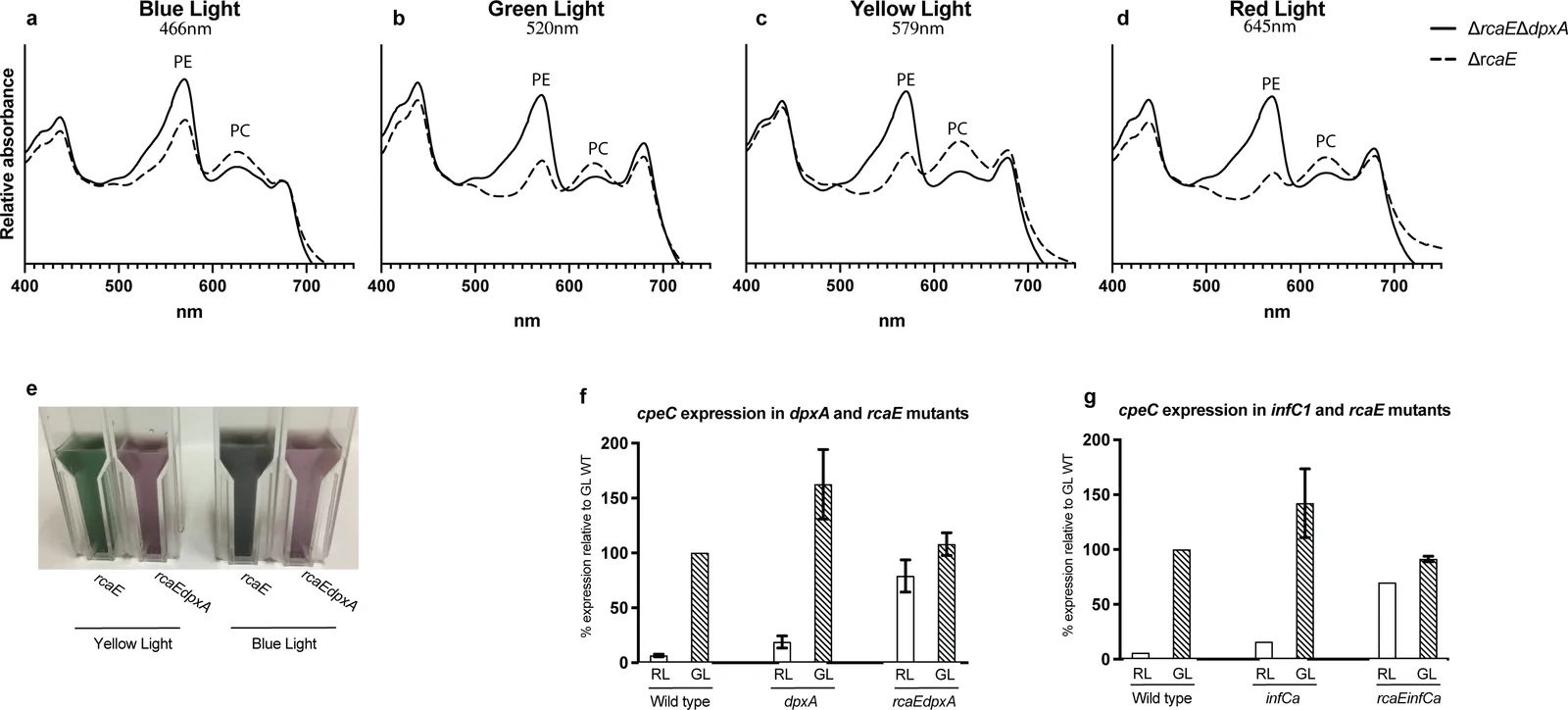
Representative whole-cell absorbance scans of Δ*rcaE* mutant Δ*rcaE*Δ*dpxA* mutant cells grown in (**a**) blue, (**b**) green, (**c**) yellow, and (**d**) red light at 12 µmol photons m^−2^ s^−1^. PE and PC pigment peaks are indicated on the diagram. Each scan is representative of at least 6 replicates during two experiments. (**e**) Representative pictures of Δ*rcaE* (left) or Δ*rcaE*Δ*dpxA* (right) cultures grown in blue or yellow light. (**f–g**) RNA blot analysis of mutants and WT cells in red and green light. Bars are normalized to green light WT, which is set at 100%.

To test the effect of RcaE in each light color, an in-frame clean deletion Δ*rcaE* mutant was grown in blue, green, yellow, or red light (Fig. 2a through d). Visibly, the Δ*rcaE* mutant was greenish-black in all light colors, but whole-cell spectral scans revealed differences in PE levels in blue and yellow light, showing that the DpxA-controlled system was functioning to inhibit PE in yellow light in the Δ*rcaE* mutant (Fig. 2a, c, and e).

To test the combined effects of RcaE and DpxA, an in-frame double deletion mutant, Δ*rcaE*Δ*dpxA,* was generated. The double mutant was grown in blue, green, yellow, and red LED light. Δ*rcaE*Δ*dpxA* cells were brownish-red in all light colors tested, indicative of high PE and low PC, and the majority of PE regulation appeared to be abolished (Fig. 2a through d). Unlike Δ*rcaE*, which displays blue-yellow acclimation, the Δ*rcaE*Δ*dpxA* double mutant was reddish-brown in both colors with no visible pigment regulation (Fig. 2e). Complementation of Δ*rcaE*Δ*dpxA* with *dpxA* on a plasmid restored the black phenotype, countering the possibility that the phenotype is due to a polar effect on genes downstream of *dpxA* (Fig. S1).

RNA blot analysis for the PE gene *cpeC* was conducted for wild type, Δ*dpxA*, and Δ*rcaE*Δ*dpxA* cells in red light and green light. Consistent with regulation observed at the pigmentation level, there was no observed light color regulation of these genes in the Δ*rcaE*Δ*dpxA* mutant between RL-grown and GL-grown cells (Fig. 2f). The RNA blot experiment was repeated for the Cgi pathway gene *infCa,* which we predict could be a component through which DpxA functions, and found that the pattern of RNA accumulation was similar to the observation for the *dpxA* mutant (Fig. 2g).

### Discovery of the *dpx* operon: *dpxABC*

**Fig 3:**
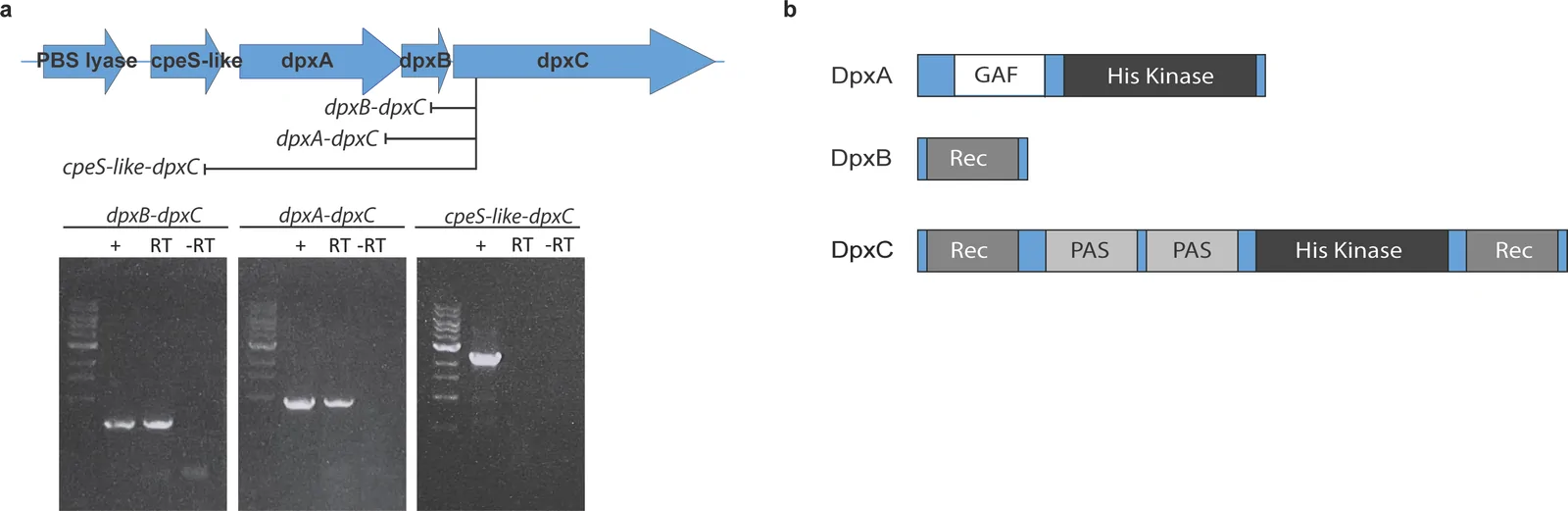
*dpxA, dpxB,* and *dpxC* are co-transcribed (**a**) Upper: Genetic structure of the region surrounding *dpxA*. Lines represent the region between genes that was amplified from cDNA produced from the RNA transcripts in wild-type cells. Lower: Agarose gels of products of RT-PCR. The first lane in each set is a DNA ladder. (+) Positive control PCR from wild-type DNA. (RT) RT-PCR conducted as described in the Materials and Methods with cDNA. (−RT) Negative control RT-PCR performed as above except without the reverse transcriptase enzyme to convert RNA to cDNA. (**b**) Predicted domain architecture of DpxA, DpxB, and DpxC proteins. Rec- Response regulator receiver; His Kinase-histidine kinase

In a mutant screen for components of the Cgi system in *rcaE*− cells, five red-brown transposon mutants (which were high in PE and low in PC in red light) were disrupted in genes located just downstream of *dpxA* (Fig. S2). Four other predicted coding sequences also have the appearance of an operon with *dpxA* (Fig. 3a). To determine if the genes were co-transcribed, reverse transcription polymerase chain reaction (RT-PCR) amplification was employed to target the intergenic regions between candidate genes. mRNA was present between *dpxA* and the two downstream genes (named *dpxB* and *dpxC*), indicating that *dpxA* is in an operon with those two genes (Fig. 3a). DpxB is a single-domain response regulator family member and DpxC is similar to multi-domain hybrid histidine kinases (Fig. 3b). No mRNA was detected between *dpxA* and its nearest upstream gene, confirming that *dpxA, dpxB,* and *dpxC* compose the entirety of the *dpx* operon.

### Regulation of chromatic acclimation by Dpx proteins

**Fig 4:**
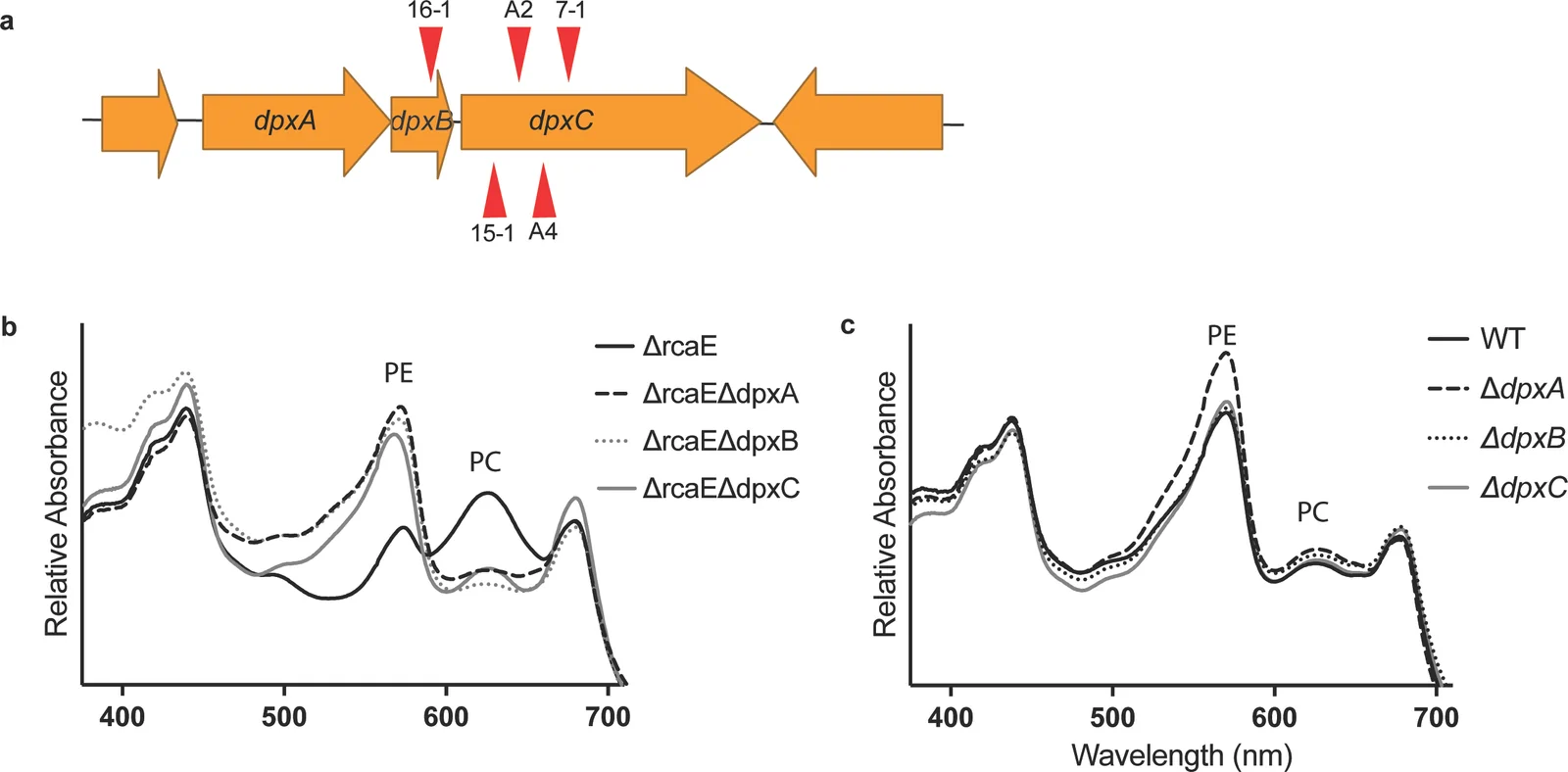
Effect of *dpxA, dpxB,* and *dpxC* in the absence of RcaE (**a**) Location of Tn5 insertion of mutants found in the *dpx* operon during a mutant screen in *rcaE*− cells. (**b**) Whole-cell absorbance scans of double deletion mutants grown in red-shifted white light. (**c**) Whole-cell absorbance scans of single deletion mutants grown in yellow light. Panel **c** is adapted from (28).

To begin to determine the roles of *dpxB* and *dpxC* in chromatic acclimation, in-frame double deletions of Δ*rcaE*Δ*dpxB* and Δ*rcaE*Δ*dpxC* were made. Both the Δ*rcaE*Δ*dpxB* and Δ*rcaE*Δ*dpxC* mutants displayed similar phenotypes to the Δ*rcaE*Δ*dpxA* mutant: they were each reddish-brown when grown in white light, with more PE and less PC than the greenish-black Δ*rcaE* mutant (Fig. 4b). Thus, all three proteins expressed from the *dpx* operon affect PE and PC levels in the absence of RcaE. Single Δ*dpxA,* Δ*dpxB,* and Δ*dpxC* mutants were created and grown in yellow light, where the effect of DpxA is most detectable. Unlike Δ*dpxA*, neither the Δ*dpxB* nor the Δ*dpxC* mutant has a pigmentation phenotype distinguishable from wild type in yellow light (Fig. 4c). Thus, DpxB and DpxC appear to mainly regulate pigment abundance in the absence of RcaE.

### The *dpx* operon is conserved across diverse cyanobacteria, likely through horizontal gene transfer

To elucidate the occurrence of the *dpx* operon, we used BLAST to probe cyanobacterial genomes for similar genes in other organisms. A list of cyanobacteria with homologous proteins to DpxA was generated. The genomic context of 95 *dpxA* homologs was examined, and 86 species had downstream genes encoding proteins similar to DpxB and DpxC (Table S2). The strains that contain the *dpx* operon were examined on previously-constructed phylogenetic trees (36) and then sorted into orders based on a recent phylogenomic and polyphasic classification system of cyanobacteria (37). The *dpx* operon was found in the highest number in the Nostocales order, which is the order to which *F. diplosiphon* is a member (Table 2). Outside of Nostocales, species with the *dpx* operon also were members of 12 other orders of cyanobacteria: Acaryochloridales, Chroococcales, Chroococcidiopsidales, Coleofasciculales, Gomontiellales, Leptolyngbyales, Nodosilineales, Oculatellales, Oscillatoriales, Pleurocapsales, Pseudanabaenales, Synechococcales (Table 2). The orders of cyanobacteria that contain the *dpx* operon are not the most closely related, with some being distantly related (37), indicating a diverse set of cyanobacteria phylogenetically, but also possibly in physiological capabilities.

**TABLE 2:** Phylogenetic distribution of the *dpx* operon by order, as designated in a Strunecký et al. (37) Number of genera were estimated from information found in AlgaeBase (38) and CyanoDB (39)

Order	Approx. genera in order	Genera or families associated with *dpx* operon identified	# species with *dpx* identified
Acaryochloridales	~7	Acaryochloridaceae (Family)	1
Aegeococcales	1	None	0
Chroococcales	~50	Chlorogloea, Gloeocapsa, Gloeocapsopsis	1
Chroococcidiopsidales	~11	Chroococcidiopsis, Aliterella	10
Coleofasciculales	~25	Coleofasciculaceae (Family)	1
Desertifilales	~4	None	0
Geitlerinematales	1	None	0
Gloeobacterales	~2	None	0
Gloeomargaritales	1	None	0
Gomontiellales	~15	Cyanothece, Chamaesiphon	3
Leptolyngbyales	~25	Leptolyngbya, Alkalinema, Adonisia, Kovacikia, Leptothermofonsia	13
Nodosilineales	~35	Leptothoe	1
Nostocales	~130-150	Fremyella, Tolypothrix, Calothrix, Nostoc, Aulosira, Mojavia, Nostochopsis, Pelatocladus, Scytonema, Brasilonema, Stigonema, Brunnivagina, Aetokthonos, Altericista, Iningainema, Mastigocoleus, Dulcicalothrix	45
Oculatellales	~25	Egbenema, Elainella, Oculatellaceae (Family)	4
Oscillatoriales	~50-60	Microcoleus	1
Pleurocapsales	~10-24	Xenococcaceae (Family)	1
Prochlorotrichales	1	None	0
Pseudanabaenales	~8	Pseudanabaena, Tumidithrix	2
Spirulinales	~6	None	0
Synechococcales	~30-50	Synechocystis	1
Thermostichales	1	None	0
Unclassified	Unknown	Cyanobacteria bacterium J06639_18	1

**Fig 5:**
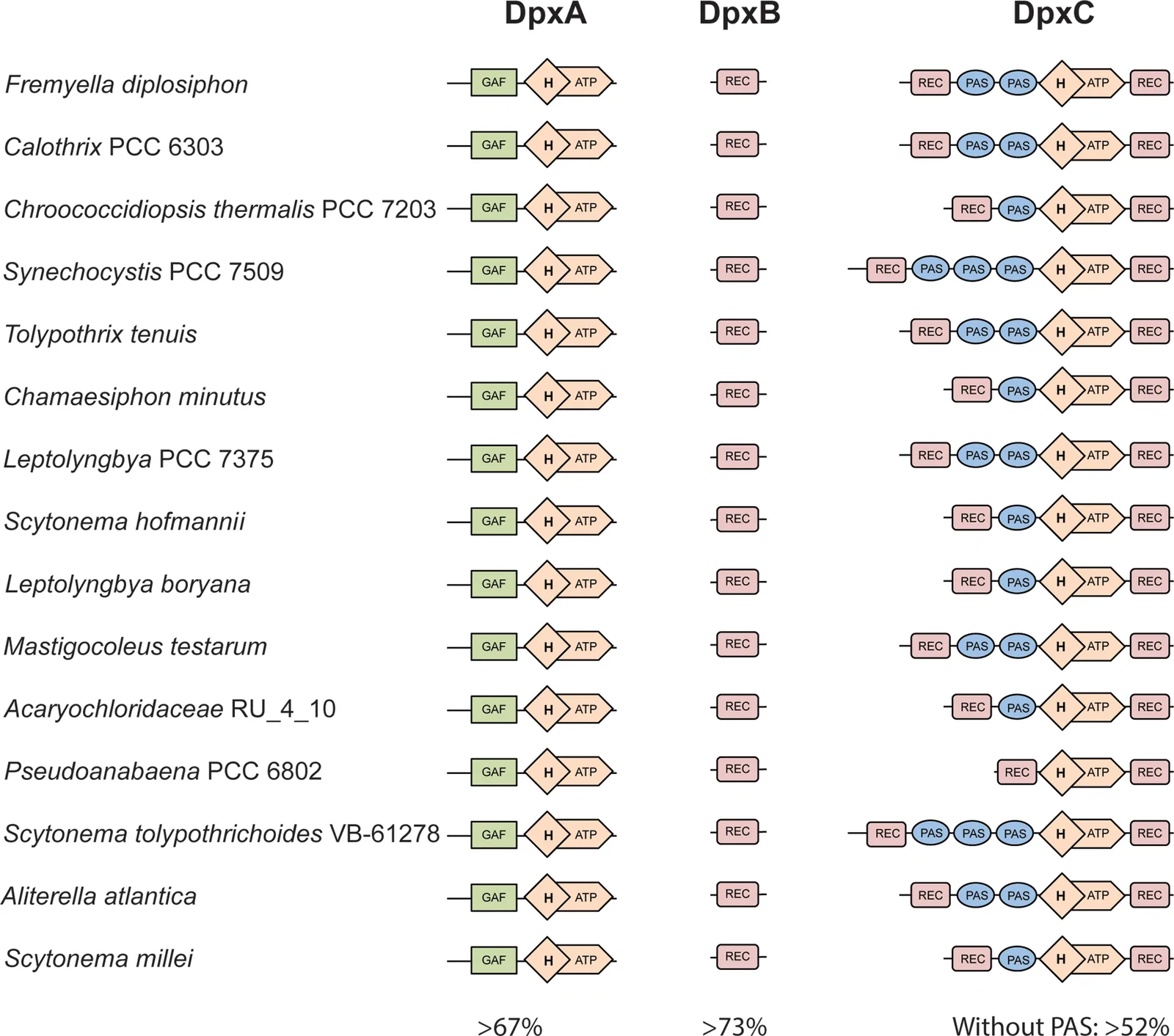
Conservation of *dpx* operon. Domain architecture of representative species with operons similar to the *dpx* operon. Receiver domains are indicated by pink boxes labeled REC. Histidine kinase domains are indicated by conjoined orange diamonds and arrows labeled H and ATP. PAS domains are indicated by blue ovals labeled PAS. GAF domains are indicated by green rectangles labeled GAF. The lowest percent identity of each protein, calculated by pairwise comparisons, of each protein are at the bottom of each column.

**Fig 6:**
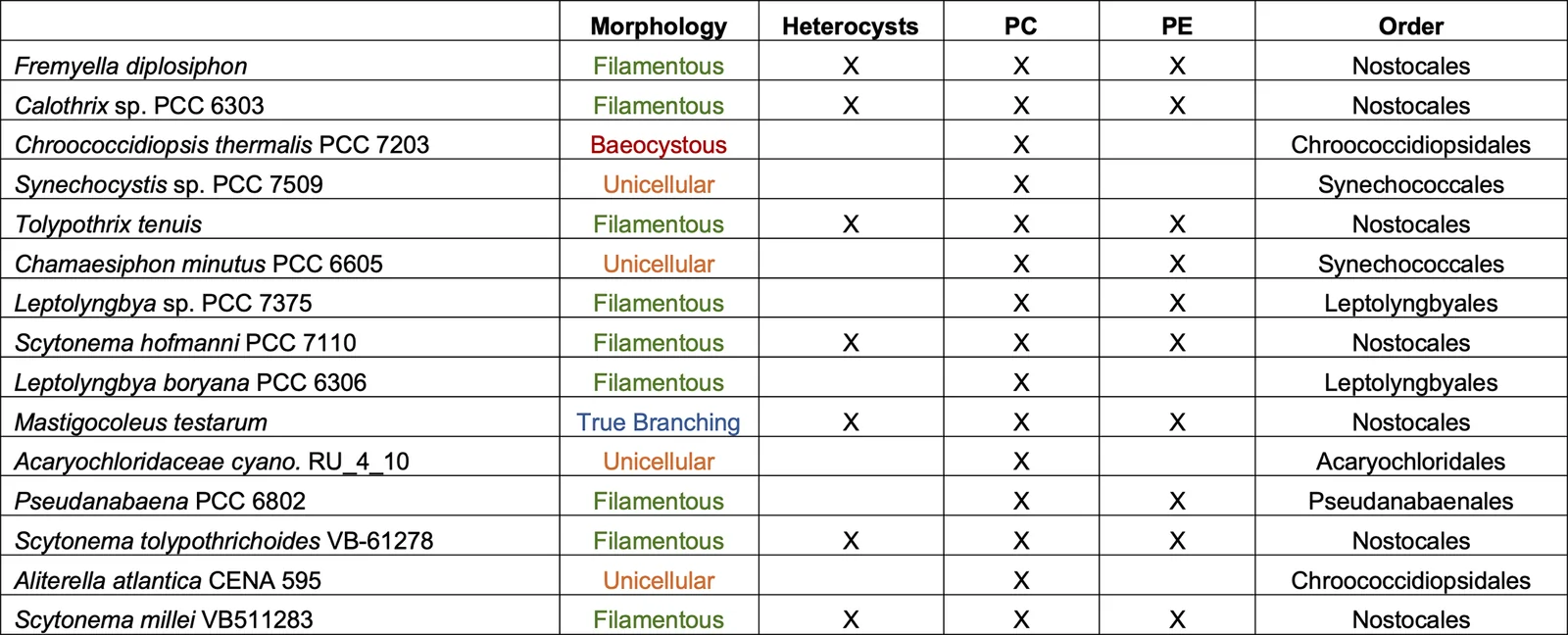
Diversity of representative species with the *dpx* operon, PC is phycocyanin, and PE is phycoerythrin.

Fifteen representative species of *dpx-*containing cyanobacteria were analyzed at a deeper level. Pairwise analysis of representative DpxA homologs were >67% identical in amino acid sequence to DpxA and DpxB homologs were at least 73% identical. DpxC proteins were also very similar, except that DpxC homologs had wide variation in the number of PAS domains (Fig. 5). The DpxC from *F. diplosiphon* has two PAS domains, but other species have DpxC homologs with zero to three PAS domains (Table S2). When the variable PAS domains are deleted from the sequence, DpxC proteins are greater than 52% identical. The representative strains were diverse in many features (Fig. 6). Notably, not all strains with the *dpx* operon express PE (Fig. 6), although the role for the *dpx* operon described in *F. diplosiphon* is related to the regulation of PE abundance.

During analysis of strains with the *dpx* operon, we discovered another operon in *F. diplosiphon* that is very similar to the *dpx* operon but with variations in the number of PAS domains in the DpxA-like protein (Fig. S3). Interestingly, unlike the *dpx* operon, this *dpx-*like operon is mainly conserved across species that are very closely related (99% of the order Nostocales), with PE or PEC and PC (Table S1). Thus, this second operon may control less diverse processes than the original *dpx* operon, although no function(s) for this operon have been determined. The conservation of the *dpx*-like operon serves as a comparison group with the *dpx* operon, as the *dpx-*like operon is conserved primarily in closely related species, while the *dpx* operon is also conserved in distantly related species.

## DISCUSSION

*F. diplosiphon* has been a model organism for the study of Type three chromatic acclimation since its discovery in 1902 (3, 40). Chromatic acclimation of pigmentation in *F. diplosiphon* is clearly more complex than originally thought, as it is not confined to red-green systems, since teal-yellow light acclimation is also observed (28). In this manuscript, a comparison of our newly generated mutants—specifically the Δ*rcaE*Δ*dpxA,* Δ*rcaE*Δ*dpxB*, and Δ*rcaE*Δ*dpxC* double-deletion series—with established single mutants like Δ*rcaE* and Δ*dpxA* reveals a clear functional link within the sensory network. While the Δ*rcaE* single mutant is able to respond to yellow-teal light and maintains a greenish-black appearance, the double mutants exhibit a loss of chromatic acclimation and remain constitutively reddish-brown. Furthermore, the observation that Δ*dpxB* and Δ*dpxC* phenotypes only emerge in an *rcaE-* background demonstrates that these proteins are essential for the integrated multispectral circuit rather than acting as independent parallel regulators. While interactions between cyanobacteriochromes exist (41, 42), the precise mechanistic details of how one modulates another are not well-defined (17).

We determined that DpxA is co-transcribed with two other regulatory genes: *dpxB*, which encodes a single-domain response regulator; and *dpxC*, which encodes a hybrid histidine kinase with a variable number of PAS domains. When *dpxA, dpxB,* and *dpxC* were each deleted in conjunction with deletion of *rcaE*, cells were reddish-brown in color, indicating that chromatic acclimation was disrupted. Thus, all three Dpx proteins have a role in cell pigmentation in the absence of RcaE. DpxA also represses PE levels independently of RcaE, as previously demonstrated (28). The observation that DpxB and DpxC phenotypes are contingent upon the loss of RcaE suggests that these pathways form an integrated sensory network rather than isolated, parallel pathways. Adding to the potential complexity of sensory networks, *F. diplosiphon* has 27 ORFs predicted to be cyanobacteriochromes (43), and RcaE has been shown to regulate the transcription of another cyanobacteriochrome, IflA (41). Given the large number of predicted cyanobacteriochromes in the genome and the interactions already known, there are likely to be more interactions between cyanobacteriochrome-controlled systems in *F. diplosiphon*.

DpxA is a strong candidate to be the sensor of the previously defined Cgi system. This is supported by a few lines of evidence. The most notable is the loss of regulation in red and green light in the Δ*rcaE*Δ*dpxA* double mutant. The loss of the Cgi system effect in the absence of *dpxA* strongly suggests that DpxA is a component of this system. Although DpxA responds maximally to teal and yellow light, it also displays greater activity in red light when compared to green light (28). Thus, the observation of the Cgi system’s role in red and green light may still be due to sensing by DpxA. The Cgi system’s mechanism involves post-transcriptional attenuation at the 5’ leader sequence of the *cpeC* gene (26). The translation initiation factor IF3a also functions to repress PE in an RcaE-absent, light-dependent manner, and an IF3a mutant shows a similar phenotype to DpxA in RcaE’s absence (27). RNA blots in this study indicated similar regulation patterns of repression of PE (Fig. 2f and g). This suggests that IF3a, the Cgi system, and the Dpx system may all be part of the same pathway. Future work will further clarify the mechanistic overlap between the Rca, Dpx, Cgi, and IF3a-mediated systems.

Another aspect of the interactions between RcaE and DpxA may be crosstalk between the kinases and response regulators in the Rca and Dpx systems. Bacterial two-component systems typically exhibit high specificity between cognate histidine kinases and RRs to prevent deleterious crosstalk (44). However, in the absence of the cognate histidine kinase, there are examples of kinases that can “crosstalk” with the response regulators, phosphorylating the response regulator only in the absence of its cognate kinase (45). Although not shown directly, Dpx proteins may engage in crosstalk with the response regulators of the Rca system.

Exploration of patterns of evolution of Dpx revealed that the *dpx* operon is conserved across ecologically diverse cyanobacterial species. Despite the phylogenetic distance between these species, the Dpx proteins exhibited high sequence identity, with DpxA homologs sharing greater than 67% identity, DpxB homologs greater than 73% identity, and DpxC homologs greater than 52% identity (excluding variable PAS domains). The *dpx* operon’s conservation across phylogenetically distant cyanobacterial species, with high amino acid identity, points to a fundamental cellular role. However, many of these species lack PE or phycoerythrocyanin (PEC) phycobiliproteins, despite the *dpx* operon’s role in PE/PC regulation in *F. diplosiphon*. The *dpx* operon is present in several different orders of bacteria but is only present in small numbers for most orders, which are not closely related to each other. While it is possible that the *dpx* operon is ancestral to cyanobacteria and has been lost many times, this does not appear remotely parsimonious. Instead, the evidence strongly supports the acquisition of the *dpx* operon by horizontal gene transfer and diverse roles for the operon outside of chromatic acclimation. Two-component systems in cyanobacteria mediate diverse developmental and acclimation responses, including stress, nutrient acquisition, phototaxis, and morphology (46). The *dpx* operon may thus sense and respond to other environmental cues, even in species not performing chromatic acclimation with PE. This system may also control physiological responses outside of CA3, as has been noted in other cyanobacteriochrome-controlled systems (29).

The observed variation in the number of PAS domains within DpxC homologs (zero to three) is indicative of PAS domain duplication, deletion, and shuffling. This aligns with previous reports of extensive PAS domain shuffling in cyanobacteria (47). PAS domains are ubiquitous sensory modules perceiving diverse signals, including light, redox states, and gases (48). Their modularity and recombination propensity facilitate rapid evolution of new sensory specificities (48). This high variability in PAS domains in the Dpx proteins suggests a mechanism for fine-tuning sensory input without altering core signal transduction machinery, enabling adaptation to fluctuating environmental cues. Different PAS configurations could allow the Dpx system to sense distinct wavelengths or other environmental factors, expanding the adaptive repertoire. This provides a powerful means for generating sensory diversity, crucial for cyanobacteria to colonize diverse niches.

## SUPPLEMENTAL MATERIAL

Supplemental figures and tables jb.00023-26-s0001.docx Figures S1 to S3, and Tables S1 and S2.

ASM does not own the copyrights to Supplemental Material that may be linked to, or accessed through, an article. The authors have granted ASM a non-exclusive, world-wide license to publish the Supplemental Material files. Please contact the corresponding author directly for reuse.
