# Supplementary material for: Multispectral regulation of chromatic acclimation by integration of the Rca system and the conserved dpx operon
Source: J Bacteriol. 2026 Jul 15;208(8):e00023-26. doi: 10.1128/jb.00023-26 (PMC13492139; doi:10.1128/jb.00023-26)
Supplement: Supplementary file 1 [file jb.00023-26-s0001.docx]

**Supplemental Figures and Tables**

**Figure S1.** Complementation of the Δ*rcaE*Δ*dpxA* mutant. (A) Photographs of Δ*rcaE* (left), Δ*rcaE*Δ*dpxA* (middle), and Δ*rcaE*Δ*dpxA* complemented with *dpxA* (right) (B) Whole cell absorption spectra of cultures of Δ*rcaE* (left), Δ*rcaE*Δ*dpxA* (middle), and Δ*rcaE*Δ*dpxA* complemented with *dpxA*. Scans and pictures are representative of three independent experiments. Phycoerythrin (PE) and phycocyanin (PC) peaks are labeled.

**Figure S2.** Genome map and whole cell absorbance scans of brown mutants discovered in a transposon mutant screen.

**Figure S3.** Conservation of *dpx-*like operon. Domain architecture of the proteins contained in operons similar to the *dpx* operon. Receiver domains are indicated by pink boxes labeled REC. Histidine kinase domains are indicated by conjoined orange diamonds and arrows labeled H and ATP. PAS domains are indicated by blue ovals labeled PAS. GAF domains are indicated by green rectangles labeled GAF. The lowest percent identity of each protein calculated by pairwise comparisons of each protein are at the bottom of each column.

**Table S1**. Diversity of representative species with *dpx-like* operon

**Table S2.** Species with *dpx* operon

| **Scientific Name** | **DpxA Accession #** | **DpxB Accession #** | **DpxC Accession #** | **# of PAS (DpxC)** |
| --- | --- | --- | --- | --- |
| *Fremyella diplosiphon (Tolypothrix* sp*.* 7601*)* | EKE99509.1 | EKE99508.1 | EKE99507.1 | 2 |
| unclassified *Tolypothrix* | WP_045867496.1 | WP_045867495.1 | WP_045867494.1 | 2 |
| *Tolypothrix tenuis* | WP_096576275.1 | WP_096576273.1 | WP_096576271.1 | 2 |
| *Nostoc linckia* FACHB-104 | MBD2214145.1 | MBD2214146.1 | MBD2214147.1 | 2 |
| *Calothrix membranacea* FACHB-236 | MBD2164889.1 | MBD2164888.1 | MBD2164887.1 | 2 |
| *Tolypothrix* sp. PCC 7910 | WP_167719252.1 | WP_167719255.1 | WP_167719258.1 | 2 |
| *Aulosira* sp. DedQUE10 | MDZ7959151.1 | MDZ7959150.1 | MDZ7959149.1 | 2 |
| *Calothrix* | WP_190545490.1 | WP_190545597.1 | WP_190545488.1 | 2 |
| unclassified *Calothrix* | WP_190635430.1 | WP_190635514.1 | WP_190635431.1 | 2 |
| *Tolypothrix* sp. FACHB-123 | WP_190742579.1 | WP_190742868.1 | WP_190742577.1 | 2 |
| *Calothrix* sp. NIES-2098 | WP_096586584.1 | WP_096586583.1 | WP_096586582.1 | 2 |
| *Aulosira* sp. ZfuCHP01 | MDZ8053591.1 | MDZ8053590.1 | MDZ8053589.1 | 2 |
| *Nostoc* sp. T09 | WP_086687560.1 | WP_086687561.1 | WP_086687562.1 | 2 |
| *Calothrix* sp. NIES-2100 | WP_096603638.1 | WP_096603637.1 | WP_096603636.1 | 2 |
| *Nostoc* sp. 106C | WP_086767985.1 | WP_445253142.1 | WP_086767989.1 | 2 |
| *Nostoc* sp. RF31YmG | OUL18791.1 | OUL18792.1 | OUL18793.1 | 2 |
| *Calothrix* sp. PCC 6303 | WP_015195961.1 | WP_015195963.1 | WP_015195964.1 | 2 |
| *Mojavia pulchra* JT2-VF2 | MBW4561871.1 | MBW4561872.1 | MBW4561873.1 | 2 |
| *Nostochopsis* sp. | MGJ5672730.1 | MGJ5672729.1 | MGJ5672728.1 | 2 |
| *Pelatocladus* sp. BLCC-F211 | WP_374872436.1 | WP_374872438.1 | WP_374872440.1 | 2 |
| *Scytonema hyalinum* WJT4-NPBG1 | MBW4500437.1 | MBW4500438.1 | MBW4500439.1 | 2 |
| *Brasilonema* sp. UFV-L1 | WP_169217935.1 | WP_169217934.1 | WP_211175989.1 | 3 |
| *Scytonema* sp. PCC 10023 | WP_414586023.1 | WP_414586024.1 | WP_414586025.1 | 2 |
| *Stigonema ocellatum SAG 48.90 = DSM 106950* | MBR8833412.1 | MBR8833413.1 | MBR8833414.1 | 2 |
| *Scytonema sp. PRP1* | WP_332993135.1 | WP_073630224.1 | WP_442994235.1 | 2 |
| *Scytonema* sp. HK-05 | WP_073630222.1 | WP_073630224.1 | WP_073630226.1 | 2 |
| *Scytonema tolypothrichoides* VB-61278 | KAB8331417.1 | KAB8331416.1 | KAB8331748.1 | 3 |
| *Brasilonema bromeliae* | WP_169156621.1 | WP_169156620.1 | WP_246275696.1 | 3 |
| *Elainellaceae cyanobacterium* | GAB4368936.1 | GAB4368930.1 | GAB4368925.1 | 1 |
| *Brunnivagina elsteri* | WP_095722240.1 | WP_095722239.1 | WP_095722238.1 | 2 |
| *Scytonema hofmannii* | WP_017747799.1 | WP_017747798.1 | WP_017747797.1 | 1 |
| unclassified *Nostoc* | WP_190705834.1 | WP_190705832.1 | WP_190705829.1 | 2 |
| *Aetokthonos hydrillicola* | WP_208349430.1 | WP_390691849.1 | WP_208349423.1 | 1 |
| *Calothrix* sp. CSU_2_0 | NJR16086.1 | NJR16085.1 | NJR16084.1 | 2 |
| *Xenococcaceae cyanobacterium* | MGL5871098.1 | MGL5871097.1 | MGL5871096.1 | 1 |
| *Altericista* sp. CCNU0014 | WP_404783822.1 | WP_404783820.1 | WP_404783818.1 | 1 |
| *Calothrix* sp. NIES-4101 | BAZ42929.1 | BAZ42930.1 | BAZ42931.1 | 2 |
| *filamentous cyanobacterium* CCP2 | PSB20466.1 | PSB20467.1 | PSB20468.1 | 1 |
| *Aliterella atlantica* | WP_045052634.1 | WP_045052633.1 | WP_045052632.1 | 2 |
| *Chroococcidiopsis* sp. | WP_438553452.1 | WP_438553453.1 | WP_438553454.1 | 1 |
| *Calothrix* sp. UHCC 0171 | WP_323341016.1 | WP_323341017.1 | WP_416336321.1 | 2 |
| *Iningainema tapete* | WP_190830977.1 | WP_190830981.1 | WP_190830984.1 | 1 |
| cyanobacterium TDX16 | OWY66196.1 | OWY66195.1 | OWY66194.1 | 1 |
| *Acaryochloridaceae cyanobacterium* RU_4_10 | NJM76994.1 | NJM76995.1 | NJM76996.1 | 1 |
| *Scytonema* sp. UIC 10036 | WP_155750371.1 | WP_155750370.1 | WP_155750369.1 | 2 |
| *Scytonema* sp. NUACC26 | WP_407899543.1 | WP_407899541.1 | WP_407899539.1 | 2 |
| *Calothrix* sp. C42_A2020_038 | MBF2063226.1 | MBF2063225.1 | MBF2063224.1 | 1 |
| *Mastigocoleus testarum* | WP_027845155.1 | WP_027845154.1 | WP_027845153.1 | 2 |
| *Mastigocoleus* sp. MO_188.B34 | WP_300636349.1 | WP_300636347.1 | WP_300636345.1 | 2 |
| *Microcoleaceae cyanobacterium* | MGL5081682.1 | MGL5081683.1 | MGL5081684.1 | 4 |
| *Cyanobacteria bacterium* J06639_18 | MEO0968771.1 | MEO0968770.1 | MEO0968769.1 | 2 |
| *Chroococcidiopsis* sp. SAG 2025 | WP_317106182.1 | WP_317106181.1 | WP_317106180.1 | 1 |
| *Leptolyngbya ohadii* | WP_088889133.1 | WP_088889134.1 | WP_088889135.1 | 1 |
| *Leptolyngbya* | WP_190652229.1 | WP_017291473.1 | WP_017291472.1 | 1 |
| *Leptolyngbya* sp. GGD | WP_268242098.1 | WP_017291473.1 | WP_017291472.1 | 1 |
| *Leptolyngbya boryana* | WP_316429077.1 | WP_017291473.1 | WP_017291472.1 | 1 |
| *Leptolyngbya* | WP_017291474.1 | WP_017291473.1 | WP_017291472.1 | 1 |
| *Egbenema* sp. FJBBOT/2019 | WP_416675993.1 | WP_416675988.1 | WP_416675998.1 | 1 |
| *Elainella* sp. Prado103 | MCU0527358.1 | MCU0527357.1 | MCU0527356.1 | 1 |
| *Leptolyngbya* sp. Prado105 | MCU0550745.1 | MCU0550744.1 | MCU0550743.1 | 1 |
| *Chroococcidiopsis* | WP_015154448.1 | WP_015154449.1 | WP_106166391.1 | 1 |
| *Scytonema millei* | WP_039716602.1 | WP_039716601.1 | WP_039716600.1 | 1 |
| *Chroococcidiopsis* sp. CCALA 051 | WP_106546800.1 | WP_015154449.1 | WP_106546801.1 | 1 |
| *Chroococcidiopsidales cyanobacterium* LEGE 13417 | MBE9019912.1 | MBE9019913.1 | MBE9019914.1 | 1 |
| *Chroococcidiopsis thermalis* PCC 7203 | WP_015154448.1 | WP_015154449.1 | WP_015154450.1 | 1 |
| *Synechocystis* sp. PCC 7509 | WP_009632723.1 | WP_009632724.1 | WP_009632725.1 | 3 |
| *Chroococcidiopsis* sp. CCNUC1 | WP_250016865.1 | WP_015154449.1 | WP_250016866&7.1 | 1 |
| *Oculatellaceae cyanobacterium* Prado106 | MCU0569470.1 | MCU0569469.1 | MCU0569468.1 | 1 |
| *Chroococcidiopsis* sp. [FACHB-1243] | WP_192161120.1 | WP_015154449.1 | WP_192161121.1 | 1 |
| *Calothrix* sp. HK-06 | OKH41305.1 | OKH41304.1 | OKH41303.1 | 1 |
| *Chlorogloea* sp. CCALA 695 | WP_106369330.1 | WP_106369329.1 | WP_106369328.1 | 3 |
| *Pseudanabaena* sp. PCC 6802 | WP_019499258.1 | WP_019499259.1 | WP_019499260.1 | 0 |
| unclassified *Leptolyngbya* | WP_430681061.1 | WP_430681060.1 | WP_430681059.1 | 1 |
| *Chamaesiphon minutus* | WP_015159580.1 | WP_015159581.1 | WP_015159582.1 | 1 |
| *Chamaesiphon polymorphus* | WP_106310561.1 | WP_106310554.1 | WP_245894342.1 | 1 |
| *Leptolyngbya* sp. PCC 7375 | EKV00511.1 | EKV00510.1 | EKV00509.1 | 2 |
| *Adonisia turfae* | WP_163670196.1 | WP_163670198.1 | WP_163700542.1 | 2 |
| *Chamaesiphon* sp. OTE_75_metabat_556 | WP_309736776.1 | WP_309736775.1 | WP_309736774.1 | 1 |
| *Leptolyngbya ectocarpi* | WP_193994734.1 | WP_193994735.1 | WP_193994736.1 | 2 |
| *Pseudanabaenaceae cyanobacterium* LEGE 13415 | MBE9010987.1 | MBE9010988.1 | MBE9010989.1 | 1 |
| *Leptothoe* sp. PORK10 BA2 | WP_323364703.1 | WP_323364704.1 | WP_323364705.1 | 2 |
| *Alkalinema* sp. CACIAM 70d | OUC12217.1 | OUC12216.1 | OUC12215.1 | 1 |
| *Coleofasciculaceae cyanobacterium* | HEY9639516.1 | HEY9639517.1 | HEY9639518.1 | 3 |
| *Alkalinema pantanalense* | WP_431631787.1 | WP_431631786.1 | WP_431631785.1 | 1 |
| *Alkalinema* sp. FACHB-956 | WP_190570312.1 | WP_190570314.1 | WP_190570316.1 | 1 |
|  |  |  |  |  |
